# Predictors of Vascular Cognitive Impairment Poststroke in a Middle Eastern (Bahrain) Cohort: A Proposed Case-Control Comparison

**DOI:** 10.2196/resprot.5903

**Published:** 2016-11-28

**Authors:** Claire Donnellan, Mona Al Banna, Noor Redha, Adel Al Jishi, Isa Al Sharoqi, Safa Taha, Moiz Bakhiet, Fatema Abdulla, Patrick Walsh

**Affiliations:** ^1^School of Nursing and MidwiferyFaculty of Health SciencesUniversity of Dublin, Trinity CollegeDublinIreland; ^2^Department of Clinical NeurosciencesSalmaniya Medical ComplexSalmaniyaBahrain; ^3^Department of Molecular MedicinePrincess Al Jawhara Center for Genetics and Inherited DiseasesSalmaniyaBahrain; ^4^School of MedicineRoyal College of Surgeons in Ireland–BahrainBusaiteenBahrain

**Keywords:** stroke, cognition, vascular dementia, assessment, biomarkers, protocol

## Abstract

**Background:**

Poststroke dementia and cognitive impairment are associated with poor long-term outcomes after stroke. The contribution of genetic factors such as the presence of apolipoprotein (ApoE) ɛ4 allele and its association with cognitive impairment poststroke remains inconclusive, particularly in Middle Eastern regions.

**Objective:**

The aim of this study is to examine all correlates and potential predictors of cognitive impairment including self-awareness and regulation deficits in stroke patients and compare these functions with healthy older adults from a Middle Eastern population.

**Methods:**

A prospective stroke sample of 200 patients (case group) and 100 healthy aging individuals (control group) will be recruited from the largest medical complex in Bahrain. A neuropsychological battery of cognitive assessments (global, executive, and metacognition) will be conducted on all participants. Participants will be categorized into 4 subgroups (nonvascular cognitive impairment, vascular cognitive impairment with no dementia, vascular dementia, and mixed dementia) using standardized cognitive assessment scores and the Diagnostic and Statistical Manual of Mental Disorders, Fourth Edition, dementia criteria. Biomarkers will include ApoE genotype, soluble form of receptor for advanced glycation end products, neprilysin, beta-secretase 1, biochemistry, and hematology measurements.

**Results:**

The primary study outcome is to determine early risk factors for cognitive impairment after stroke in a Bahraini cohort. The study has received full ethical approval from the Bahrain Ministry of Health and from the affiliated university.

**Conclusions:**

With increasing stroke incidence rates in the Middle East, this research study will provide useful biological and epidemiological data for future development and planning of health policies and guidelines for stroke care within the Gulf region.

## Introduction

Stroke is becoming a major health problem in the Middle East as incidence rates are increasing comparably with western countries [1-3]. In tandem with this increasing number of new cases of stroke per annum are the growing demands and pressures impacting health systems in the region. Advances in stroke research internationally have aimed to improve patient care and quality of life while reducing the perceived burden and health care system demands. However, empirical evidence regarding the presentation of the most common consequences poststroke is currently not available in the Middle East.

Cognitive impairment is one of the most common sequelae following stroke with 40% to 75% of stroke survivors experiencing some sort of cognitive deficit [4,5]. For the majority of patients, some degree of cognitive impairment will be evident in the acute phase poststroke [6]. Predictors of cognitive impairment include type of stroke, recurrent episodes, the site and laterality of the lesion(s), volume of cerebral infarction, medial temporal lobe atrophy, and coexistent neurodegenerative pathology. Other biological factors known to exacerbate cognitive impairment further are aphasia, diabetes mellitus, atrial fibrillation, and depression [7]. Many of the cognitive problems resolve over time, but approximately 35% of individuals will be left with some residual cognitive impairment [8,9]. Poststroke dementia and cognitive impairment are associated with poor long-term outcomes, including survival and disability, up to 4 years after stroke [9,10]. There has been some investigation regarding the contribution of apolipoprotein (ApoE) ɛ4 allele [11] and its association with cognitive impairment poststroke and overall disease outcome [12-17]. However, results remain inconclusive, and further research is required in order to determine and clarify the role of ApoE ɛ4 allele in stroke incidence and outcome particularly in Middle Eastern regions.

Many studies have been performed concerning the effect of stroke on global and executive cognitive function [18-21]; however, fewer studies have been conducted on other higher-order cognitive functions such as metacognition [22,23]. The concept of metacognition consists of self-awareness, which includes knowledge about cognitive abilities and strategies, and self-regulation, which includes cognitive monitoring and cognitive control [24]. Therefore, metacognition involves conscious knowledge of cognitive processes as well as the ability to consciously monitor and regulate one’s ongoing activities while engaging in a task [25]. These higher-order cognitive functions are required in order for individuals to recognize or be aware of their deficits post–brain injury so that they are capable of selecting activities within their capability for safe and independent functioning [26]. Deficits in relation to these cognitive functions can present significant problems in terms of motivation and goal attainment as part of rehabilitative programs. Assessment and test procedures for self-regulatory functions and metacognitive processes remain limited and are currently being considered to be included as part of cognitive function tests in future protocols [27]. The aim of this study is to examine all correlates and potential predictors of cognitive impairment including self-awareness and regulation deficits in stroke patients and compare these functions with healthy older adults from a Middle Eastern population.

## Methods

### Study Design

This is a case-control study. A longitudinal quantitative approach will be conducted to examine the research objectives.

### Participants: Inclusion and Exclusion Criteria

The study will involve recruiting 2 separate sample groups. The first group, known as the case group, will include recruiting individuals within 4 weeks poststroke (see Textbox 1). The second group, known as the control group, will include recruiting healthy older adults who will be age- and gender-matched with the individuals in the case group (see Textbox 2). The case group will be recruited from the largest urban teaching hospital in Bahrain and the control group from 2 large primary health care centers within close geographical proximity to the recruiting hospital.

All consecutive admissions with a confirmed diagnosis of stroke (defined as symptoms of rapid onset lasting more than 24 hours and of presumed vascular origin reflecting a focal disturbance of cerebral function, excluding isolated impairment of higher function) [28] and in accordance with International Statistical Classification of Diseases and Related Health Problems, Tenth Revision, diagnostic criteria will be tracked for eligibility to participate in the study.

### Procedure and Measures

All stroke participants will be recruited to the study within 1 to 4 weeks poststroke and will be followed up 12 months later. Initial information collected from participants will include date of birth, gender, number of years spent in formal education, marital status, and living arrangements. Information regarding the participant’s medical history will be retrieved from the healthy participants directly through interview and for stroke participants via their medical charts. This information will include past medical history of any conditions, history of medications, date of stroke, stroke type, localization of stroke, side affected, and computer tomography (brain) and/or magnetic resonance imaging (brain) results. Case and control groups will be administered the same questionnaires with the exception of the FAST, the National Institutes of Health Stroke Scale (NIHSS), and the Checklist for Cognitive and Emotional Consequences Following Stroke (CLCE-24), which will be assessed in the stroke participants only (see Table 1).

**Selection criteria for case group.**

Inclusion criteria:

- ≥18 years of age
- Written informed consent to participate
- First-ever or recurrent stroke within 1 month of assessment
- Ability to participate in interview assessment with sufficient language (aphasia will be assessed using the shortened version of the Frenchay Aphasia Screening Test [FAST] with a cut-off score of ≥14)

Exclusion criteria:

- Transient ischemic attacks (TIAs) and related syndromes
- Nonverbal communication as a result of aphasia or as determined by a score of ≤13/20 on the shortened version of the FAST score
- Too medically unstable to participate in the study
- Formal diagnosis of prestroke vascular dementia or prestroke cognitive impairment

- Traumatic brain injury or traumatic intracranial or subarachnoid hemorrhage
- Visual or hearing impairment that would hinder participation in assessments
- Neurodegenerative disease (eg, Parkinson disease) or previously documented diagnosis of dementia

**Selection criteria for control group.**

Inclusion criteria:

- Healthy adults both male and female
- Age and gender matched with case group participants
- English or Arabic speaking

Exclusion criteria:

- Previous use of psychotropic medication
- Neurodegenerative disease (eg, Parkinson disease, dementia, or stroke)
- Previous stroke or transient ischemic attack (TIA)
- Meets cognitive impairment criteria

The expected time for each stroke participant interview, including all assessments, should take 45 to 60 minutes, and for each healthy participant, the interview should take 20 to 40 minutes. The control group will include healthy individuals with no history of psychological or mental illness (including ever having been prescribed psychotropic medication) or any neurodegenerative diseases. Therefore, this group will create a baseline to identify the normal level of cognitive function that is age- and gender-matched to the case group individuals. In order for measurement to be relevant to the Bahrain region population, questionnaires have been translated into Arabic and either the English or Arabic battery of tests will be used depending on the participant’s native tongue. All assessment tools have been translated from English to Arabic in accordance with international translation guidelines [29,30], and reliability tests (internal consistency) will be reported on all translated versions.

### Cognition Assessment

The case group will be categorized into 4 subgroups [27,31,32] based on the Mini-Mental State Examination (MMSE), Montreal Cognitive Assessment (MoCA), and Trail-Making Test (TMT) scores alongside Diagnostic and Statistical Manual of Mental Disorders, Fourth Edition, (DSM-IV) dementia criteria (see Textbox 3).

**Table 1:** Schedule of assessments and measures for case and control groups.

Generic variable name	Instrument/measure name	Case group		Control group
		4 weeks PS^a^	12 months PS	
Participant demographics	Age, gender, nationality, marital status, living situation, education and occupation, hand preference	x		x
Clinical details	Past medical history Oxfordshire Community Stroke Project classification	x x		x
	TOAST^b^ classification of stroke	x		
	Lesion location	x		
	Affected side	x		
	Length of hospital stay	x		
Stroke severity	National Institute of Health Stroke Severity Scale	x		
Aphasia screening	Frenchay Aphasia Screening Test	x		x
Global cognition	Mini-Mental State Examination	x	x	x
	Montreal Cognitive Assessment	x	x	x
Executive function	Trail-Making Test (A+B)	x	x	x
Metacognition	Metacognitive Questionnaire-30	x	x	x
	Checklist for Cognitive and Emotional Consequences Following Stroke to be completed by participant and by proxy	x	x	x
Premorbid cognitive functioning	Informant Questionnaire on Cognitive Decline in Elderly	x	x	x
Mood	Hospital Anxiety and Depression Scale	x	x	x
Activities of daily living	Barthel Index	x	x	x
Biomarkers	Lipid profile, HbA1c^c^, homocysteine levels, coagulation profile	x	x	x
	ApoE^d^ sRAGE^e^, BACE1^f^ and NEP^g^	x x	x	x x

^a^PS: poststroke.

^b^TOAST: Trial of Org 10172 in Acute Stroke Treatment.

^c^HbA1c: hemoglobin A1c.

^d^ApoE: apolipoprotein E.

^e^sRAGE: soluble form of receptor for advanced glycation end products.

^f^BACE1: beta-secretase 1.

^g^NEP: neprilysin.

### Clinical Laboratory Analysis

Blood will be drawn within 1 week of admission for the case group and after interview assessment for the control group. Blood specimens for the measurement of ApoE genotypes will be collected in edetic acid tubes. DNA will be purified from white blood cells using the MagNA Pure Compact Nucleic Acid Isolation Kit and stored at –20°C. ApoE genotype analysis will be performed by polymerase chain reaction–restriction fragment length polymorphism according to the method of Zivelin et al [33]. For the serum biomarkers (sRAGE, BACE-1 and NEP), blood will be collected in serum separator tubes, and the serum will be separated and stored at –80°C. The serum biomarkers will be measured using commercially available double sandwich enzyme-linked immunosorbent assay (ELISA) kits in conjunction with an ELISA plate reader. ApoE and serum biomarker analysis will be conducted at Princess Al-Jawhara Center for Genetics and Inherited Diseases. Routine biochemical analysis, including lipid profile, complete blood count, electrolytes, serum proteins, creatinine, homocysteine, and coagulation profile will be conducted at Salmaniya Medical Complex.

### Sample Size

The number of participants required to produce a statistically meaningful change in cognition between the stroke patients and healthy controls was calculated using the following formula by Bland [34] for the comparison of 2 independent samples. A sample of 75 or more in each group will detect a minimum effect size in cognition and biomarkers with a power of 0.8 at a significance level of .05. Therefore, the study will aim to recruit a minimum sample size of 100 for both the case and control groups. For conducting multiple regressions models, the study will adhere to the Green proposal where the minimum sample size should be greater than 50+8k, where k is equal to the number of independent variables [35]. Nonparametric statistical analysis will be conducted for the cognitive impairment groups’ comparisons as classified in Textbox 3.

**Classification of cognitive impairment.**

- Stroke with no vascular cognitive impairment:
  - MMSE score≥24 (with no education add 2 points, >80 years of age add 1 point)
  - MoCA score≥26 (add 1 point if≤12 years education)
  - TMT score: Trail A<78 seconds, Trail B<273 seconds
- Vascular cognitive impairment with no dementia:
  - Mild:
    - MMSE score 21-24
    - MoCA score 18-25
    - TMT score: Trail A>78 seconds, Trail B>273 seconds
  - Moderate:
    - MMSE score 10-20
    - MoCA score 10-17
    - TMT score: Trail A>78 seconds, Trail B>273
- Vascular dementia: classified according to the DSM-IV dementia criteria
- Mixed dementia: classified according to the DSM-IV dementia criteria

### Statistical Analysis

All independent and dependent study variables will be reported using descriptive statistics. Comparisons between variables for control and case groups will be determined using independent *t* tests, analysis of variance, and multivariate analysis of variance where multiple variables will be analyzed together. Other statistical tests will include correlational analysis including uni- and multivariate analysis using Pearson’s correlation and multiple regression. All variables will be checked for normal distribution in order to justify use of parametric statistics. Psychometric analysis will be conducted on all measures including and specifically those that are translated from English to Arabic (Metacognitive Questionnaire-30 and Hospital Anxiety and Depression Score). Nonparametric tests will be conducted for subgroup analysis where group sizes may be small using Spearman’s correlation and Wilcoxon tests.

### Estimated Study Outcomes

The effects of stroke will be determined by assessing all cognitive functions. The relationship between stroke severity and metacognitive functioning will be determined as will the association between cognitive impairment in the acute phase poststroke and the presence of ApoE and other biomarkers in a Middle Eastern cohort. Arabic versions of cognitive assessment tools will be validated.

### Study Organization and Funding

The study protocol has been approved by the Ministry of Health, Kingdom of Bahrain, and the Royal College of Surgeons in Ireland–Bahrain (RCSI-Bahrain) research ethics committees. Research funding was awarded for this study from the RCSI-Bahrain (grant number BR00021). Written informed consent will be obtained from all participants willing to take part; they will be informed of the right to withdraw from the study at any time.

## Results

See Table 2 for the baseline characteristics of the case and control group samples to date.

**Table 2:** Baseline characteristics of the first 51 stroke patients and 49 controls.

Baseline characteristics		Stroke	Healthy controls
Age in years, mean (SD)		59.33 (13.93)	57.51 (8.14)
**Gender, n (%)**			
	Male	40 (78)	26 (53)
**Nationality, n (%)**			
	Bahraini	31 (61)	46 (94)
	Non-Bahraini	20 (39)	3 (6)
**Living arrangement, n (%)**			
	Living alone	11 (22)	0 (0)
	Living with others—family/friends	32 (63)	46 (94)
	Other	8 (15)	3 (6)
**Education, n (%)**			
	Primary	12 (23)	6 (12)
	Secondary	20 (39)	22 (45)
	Third level	10 (19)	14 (29)
	Illiterate	9 (19)	7 (14)
**Occupation, n (%)**			
	Manual	16 (31)	5 (10)
	Nonmanual/self-employed	16 (31)	10 (20)
	Unemployed/retired	19 (38)	34 (69)
**History, n (%)**			
	TIA^a^	6 (12)	0 (0)
	Stroke	9 (17)	0 (0)
	Cardiovascular disease	9 (17)	6 (12)
	Atrial fibrillation	5 (10)	4 (8)
	Hypertension	32 (63)	27 (55)
	Diabetes	20 (39)	22 (45)
	Hyperlipidemia	19 (38)	24 (49)
	Psychiatric conditions	1 (2)	0 (0)
**Lesion location, n (%)**			
	Right hemispheric	26 (51)	—
	Left hemispheric	20 (39)	—
	Brainstem	5 (10)	—
	Unknown	0 (0)	—
**Stroke subtype, n (%)**			
	Ischemic	43 (84)	—
	Hemorrhagic, intracerebral	8 (16)	—
	Hemorhhagic, subarachnoid	0 (0)	—
**OCSP^b^ classification, n (%)**			
	Total anterior circulation	4 (8)	—
	Partial anterior circulation	18 (35)	—
	Lacunar	21 (41)	—
	Posterior circulation	8 (16)	—
**TOAST^c^ classification, n (%)**			
	Large artery atherosclerosis	14 (28)	—
	Cardioembolic	2 (4)	—
	Small artery occlusion	24 (48)	—
	Determined or undetermined etiology	11 (20)	—
**NIHSS^d^, mean (SD)**		5.77 (5.01)	—
	0-4, n (%)	20 (39)	—
	5-15, n (%)	30 (59)	—
	≥16, n (%)	1 (2)	—
**Cognitive status at baseline**			
	MMSE^e^, mean (SD)	24.09 (4.81)	27.86 (2.42)
	MoCA^f^, mean (SD)	19.78 (6.64)	25.49 (3.60)
	MCQ-30^g^, mean (SD)	62.42 (14.29)	63.2 (15.60)
	Barthel Index, mean (SD)	69.00 (31.07)	99.49 (2.10)
	Independent, n (%)	13 (25)	49 (100)
	Dependent, n (%)	38 (75)	—

^a^TIA: transient ischemic attack.

^b^OCSP: Oxfordshire Community Stroke Project.

^c^TOAST: Trial of Org 10172 in Acute Stroke Treatment.

^d^NIHSS: National Institute of Health Stroke Severity Scale.

^e^MMSE: Mini-Mental State Examination.

^f^MoCA: Montreal Cognitive Assessment.

^g^MCQ-30: Metacognitive Questionnaire-30.

## Discussion

Advances in stroke research internationally have aimed to improve patient care and quality of life and reduce perceived burden and demands on health care systems. In terms of advancing the research agenda for the management of stroke in the Middle East, some strategic approaches may be considered from previous research reviews and studies conducted. Previous research studies and reviews have highlighted core aspects relevant for stroke management in terms of the adaptation process [36,37], clinical assessment, and adherence to evidence-based practice [38,39]. Therefore, moving the stroke research agenda forward in a region such as Bahrain requires stroke studies based on potential frameworks [40,41] that can address stroke management in the context of specific regional preferences. The aim of this research study is to investigate a very current topic in the field of stroke research regarding identifying the main correlates and predictors, including biological markers, for detecting those patients most at risk for developing cognitive impairment poststroke. This research study, in addition to addressing a relatively novel research area such as metacognition, will also record the clinical and demographical profile of stroke patients. Identifying core risk factors [42] in Bahrain will provide useful epidemiological data for future development and planning of health policies and guidelines for the overall prevention [43-45], management, and delivery of stroke care. These outcomes will aid in developing stroke management and rehabilitation within a contextual framework by tailoring the population’s health needs based on specific cultural variations. Findings from these type of cognition studies will also inform future developments required for cognitive training interventions [46] specific for stroke patients and other populations with cognitive deficits as a result of neurological pathologies living in Middle Eastern countries. Finally this study will aim to ascertain recruitment considerations [47] for both healthy older adults and stroke patients who are at risk of developing dementia in this particular region.

## Abbreviations

ApoE: apolipiprotein
BACE-1: beta-secretase 1
CLCE-24: Checklist for Cognitive and Emotional Consequences Following Stroke
DSM-IV: Diagnostic and Statistical Manual of Mental Disorders, Fourth Edition
ELISA: enzyme-linked immunosorbent assay
FAST: Frenchay Aphasia Screening Test
MCQ-30: Metacognitive Questionnaire-30
MMSE: Mini-Mental State Examination
MoCA: Montreal Cognitive Assessment
NEP: neprilysin
NIHSS: National Institutes of Health Stroke Scale
OCSP: Oxfordshire Community Stroke Project
RCSI-Bahrain: Royal College of Surgeons in Ireland–Bahrain
sRAGE: soluble form of receptor for advanced glycation end products
TIA: transient ischemic attack
TMT: Trail-Making Test
TOAST: Trial of Org 10172 in Acute Stroke Treatment
